# Secure Adaptive Topology Control for Wireless Ad-Hoc Sensor Networks

**DOI:** 10.3390/s100201251

**Published:** 2010-02-03

**Authors:** Ching-Tsung Hsueh, Yu-Wei Li, Chih-Yu Wen, Yen-Chieh Ouyang

**Affiliations:** Department of Electrical Engineering, Graduate Institute of Communication Engineering, National Chung Hsing University, Taichung 402, Taiwan; E-Mails: d9764103@mail.nchu.edu.tw (C.-T.H.); g9364106@mail.nchu.edu.tw (Y.-W.L.); ycouyang@nchu.edu.tw (Y.-C.O.)

**Keywords:** secure communication protocols, adaptive topology control, sensor networks, quarantine region

## Abstract

This paper presents a secure decentralized clustering algorithm for wireless ad-hoc sensor networks. The algorithm operates without a centralized controller, operates asynchronously, and does not require that the location of the sensors be known a priori. Based on the cluster-based topology, secure hierarchical communication protocols and dynamic quarantine strategies are introduced to defend against spam attacks, since this type of attacks can exhaust the energy of sensor nodes and will shorten the lifetime of a sensor network drastically. By adjusting the threshold of infected percentage of the cluster coverage, our scheme can dynamically coordinate the proportion of the quarantine region and adaptively achieve the cluster control and the neighborhood control of attacks. Simulation results show that the proposed approach is feasible and cost effective for wireless sensor networks.

## 1. Introduction

Sensor networks are typically characterized by limited power supplies, low bandwidth, small memory sizes and limited energy [1, 2]. Thus the resource-starved nature of sensor networks poses great challenges for security, since wireless networks are vulnerable to security attacks due to the broadcast nature of the transmission medium [2–15].

In most sensor network applications, the lifetime of sensor nodes is an important concern, which can shorten rapidly under spam attacks. Moreover, maintaining network connectivity is crucial to provide reliable communication in wireless ad-hoc networks. In order not to rely on a central controller, clustering is carried out by adaptive distributed control techniques. To this end, the *Secure Adaptive Distributed Topology Control Algorithm (SADTCA)* aims at topology control and performs secure self-organization in four phases: (I) Anti-node Detection, (II) Cluster Formation, (III) Key Distribution; and (IV) Key Renewal, to protect against spam attacks.

In Phase I, in order to strengthen the network against spam attacks, the secure control is embedded into the SADTCA. A challenge is made for all sensors in the field such that normal nodes and anti-nodes can be differentiated. In Phase II, based on the operation in Phase I, the normal sensors may apply the adaptive distributed topology control algorithm (ADTCA) from [16] to partition the sensors into clusters. In Phase III, a simple and efficient key distribution scheme is used in the network. Two symmetric shared keys, a cluster key and a gateway key, are encrypted by the pre-distributed key and are distributed locally. A cluster key is a key shared by a clusterhead and all its cluster members, which is mainly used for securing locally broadcast messages. Moreover, in order to form a secure inter-cluster communication channel, a symmetric shared key may be used to encrypt the sending messages between the gateways of adjacent clusters. Since using the same encryption key for extended periods may incur a cryptanalysis risk, in Phase IV, key renewing may be necessary for protecting the sensor network and guarding against the adversary getting the keys.

Built upon the cluster-based network topology, three quarantine methods, Method 1: quarantine for clusters, Method 2: quarantine for nodes, and Method 3: quarantine for infected areas, are proposed for dynamically determining the quarantine region. In order to explore the fundamental performance of the SADTCA scheme, an analytical discussion and experiments are presented to investigate the energy consumption, communication complexity, the increase of communication overheads for data dissemination, and the percentage of the quarantine region in the sensing field when facing the spam attack.

The organization of this paper is as follows: In Section 2., we briefly introduce the related work and summary of security issues for wireless sensor network environment. Section 3. describes the system model and algorithm for secure self-organization in a cluster-based network topology. Section 4. presents dynamic approaches for determining the quarantine region. In Section 5., we analyze the SADTCA and make comparisons with protocols in the flat-based topology. In Section 6., the simulation results are shown and discussed. Finally, Section 7. draws conclusions and shows future research directions.

## 2. Literature Review

There are many vulnerabilities and threats to wireless sensor networks. The broadcast nature of the transmission medium incurs various types of security attacks. Different schemes to detect and defend against the attacks are proposed in [2–15]. A number of anti-nodes deployed inside the sensing field can originate several attacks to interfere with message transmission and even paralyze the whole sensor network. Most network layer attacks against sensor networks fall into one of the following categories:

- Acknowledgement spoofing.
- Selective forwarding.
- Sybil attacks.
- Wormholes attacks.
- Sinkhole attacks.
- *Hello* flood attacks.

The spam attack, which is a kind of flooding Denial of Service (DoS) attack, can be carried out by the anti-node inside the sensor network. Such attack can retard the message transmission and exhaust the energy of a sensor node by generating spam messages frequently. In [17] and [18], the authors propose detect and defend spam (DADS) scheme and quarantine region scheme (QRS) to address the following issues: spam detection, quarantined nodes determination, messages authentication, and quarantine region cancelation. Two detection mechanisms against spam attacks on sensor networks are proposed in DADS [17]. The first method is to filter incoming messages according to their contents and detect the nodes that send faulty messages frequently. The second method uses the frequencies of messages sent by the sensors in the same region. In DADS, the anti-node is detected by the sink, not by the sensor node. The packets of each sensor are counted by the sink. Such centralized detection architecture can be well suitable to a small-scale sensor network, but the total number of packets could be large in a large-scale sensor network. Based on the distributed strategy, the QRS makes each node to detect neighbor anti-node individually [18]. By requesting authenticated messages, each sensor node decides whether there is an anti-node in its transmitting range or not. Comparing to the central detection architecture, the total packet number is reduced rapidly and the limitation of scalability is eliminated.

These schemes classify the transmission range of the anti-node as the “quarantine region”. The nodes in the quarantine region are called “quarantined nodes”. A message must be authenticated in the quarantine regions. Any unauthenticated messages from nodes in the quarantined region will not be replied and are discarded. The nodes outside the quarantine regions do not need authentication to transmit a message even if the message was an originally authenticated message coming from a quarantine region. By this partial authentication strategy, the cost of authentication is reduced effectively.

Notice that the overheads of authentication in [17, 18] are dependent on the number of anti-nodes and the area of quarantine region. Moreover, when determining the quarantine region, the location information of the nodes is required for the approaches in [17, 18]. In contrast, based on a cluster-based topology, the proposed SADTCA adaptively forms the quarantine region without using network location information. Therefore, a management scheme, such as hierarchical clustering, may be added to further enhance the formation, message transmission, and management of a quarantine region. Our previous works [16, 19] propose the extensive research for distributed cluster-based topology control. In these algorithms, a cluster is suitable for a base unit of quarantine region such that the complexity of management of quarantine region can be reduced.

Accordingly, in this paper, the cluster-based architecture efficiently assists in forming and managing quarantine regions and effectively protects the network from attacks. Compared with [17, 18], our protocol can be used to enhance the control of quarantine region, as well as to restrain the packet number of transmitting messages caused by the anti-node. The performance comparison of the SADTCA and DADS is further investigated in Sections 5. and 6.

## 3. Secure Adaptive Distributed Topology Control Algorithm

In this section we present a secure adaptive distributed topology control algorithm (SADTCA) for wireless sensor networks. The proposed algorithm organizes the sensors in four phases: Anti-node Detection, Cluster Formation, Key Distribution, and Key Renewal. The main keys used in the network are (a) Pre-distributed Key, (b) Cluster Key, and (c) Gateway Key. Each sensor is pre-distributed with three initial symmetric keys, an identification message, and a key pool. Pre-distributed key is established with key management schemes [6, 7], and is used for anti-node detection and cluster formation in Phases I and II. The Cluster Key and Gateway Key are used for key distribution in Phase III. The key pool is used for key renewing in Phase IV. Note that since our research aims at network topology control, the pre-distributed key establishment is beyond the scope of this paper.

### 3.1. Phase I: Anti-node Detection

In order to strengthen the network against spam attacks, the secure control is embedded into the SADTCA. An authenticated broadcasting mechanism, such as the *μ*TESLA in SPINS [20], may be applied in this phase. In the authenticated broadcasting mechanism, a challenge is made for all sensors in the field such that normal nodes and anti-nodes can be differentiated. The challenge is that when a sensor broadcasts a *Hello* message to identify its neighbors, it encrypts the plaintext and then broadcasts; when receiving the *Hello* message, the sensor decrypts it. If the sensor decrypts the received message successfully, the sender is considered normal. Otherwise, the sender is said to be an anti-node. Therefore, we keep on the network topology without anti-nodes in order to make the network safe.

If an anti-node is presented in the first deployment of a sensor network, its neighboring normal nodes will notice the existence of the anti-node, since the anti-node will fail in authentication. Thus, referring to the cluster-based topology formed in Phase II, the spam attacks can be handled by adaptively forming the quarantine region as detailed in Section 4..

Notice that an external attack can be prevented by the operation of Phase I. In this work, we do not have a lightweight countermeasure to defend against authenticated malicious nodes. If the authenticated node is compromised and perform malicious activities, a mechanism for evicting the compromised nodes is required [7].

### 3.2. Phase II: Cluster Formation

When sensors are first deployed, the adaptive distributed topology control algorithm (ADTCA) from [16] may be used to partition the sensors into clusters. The following subsections overview the mechanisms of the ADTCA scheme for cluster formation.

#### Clusterhead Selection

Each sensor sets a random waiting timer, broadcasts its presence via a ‘*Hello*’ signal, and listens for its neighbor’s ‘*Hello*.’ The sensors that hear many neighbors are good candidates for initiating new clusters; those with few neighbors should choose to wait. By adjusting randomized waiting timers, the sensors can coordinate themselves into sensible clusters, which can then be used as a basis for further communication and data processing.

Sensors update their neighbor information (*i.e.*, a counter specifying how many neighbors it has detected) and decrease the random waiting time based on each ‘new’ *Hello* message received. This encourages those sensors with many neighbors to become clusterheads. The updating formula for the random waiting time of sensor *i* is:

(1) $${\mathrm{WT}}_{i}^{\left(k+1\right)}=\gamma \cdot {\mathrm{WT}}_{i}^{\left(k\right)}$$

where ${\mathrm{WT}}_{i}^{\left(k\right)}$ is the waiting time of sensor *i* at time step *k* and 0 *<* γ *<* 1 is inversely proportional to the number of neighbors. Therefore, if the timer expires, then the sensor declares itself to be a clusterhead, a focal point of a new cluster. However, events may intervene that cause a sensor to shorten or cancel its timer. For example, whenever the sensor detects a new neighbor, it shortens the timer. On the other hand, if a neighbor declares itself to be a clusterhead, the sensor cancels its own timer and joins the neighbor’s new cluster.

After applying the ADTCA, there are three different kinds of sensors: (1) the clusterheads (2) sensors with an assigned cluster ID (3) sensors without an assigned cluster ID, which will join any nearby cluster after τ seconds and become 2-hop sensors, where τ is a constant chosen to be larger than all of the waiting times. In this phase, each sensor initiates 2 rounds of local flooding to its 1-hop neighboring sensors, one for broadcasting sensor ID and the other for broadcasting cluster ID, to select clusterheads and form 2-hop clusters. Hence, the time complexity is *O*(2) rounds. Thus, the topology of the ad-hoc network is now represented by a hierarchical collection of clusters. The procedures of initial cluster formation are summarized in Table 1.

#### Gateway Selection

Observe that the clustering scheme induces non-overlapping clusters. Accordingly, to interconnect two adjacent non-overlapping clusters, one cluster member from each cluster must become a gateway. This subsection presents a method of choosing distributed gateways for adjacent non-overlapping clusters. Random waiting times and local information are applied to select gateways and further achieve communication between clusters. The result of the Phase II processing is that each cluster *i* assigns a single member to communicate with each nearby cluster *j*. The waiting timers help to ensure that the chosen member is one of the nearest members even though the topology of the system is unknown. If the clusters are too far apart (outside the range of communication *R*), no gateway sensors will be assigned.

According to the process of cluster formation, sensors can obtain local information and know the number of neighboring sensors in adjacent clusters. Therefore, given the local information, sensors may initialize their counters for gateway selection. The initialization process is summarized in Table 2. Based on the counter, clusterheads broadcast messages to trigger the gateway selection process. After applying the procedure for determining gateways, the gateway nodes broadcast messages to update the connectivity information and activate the linked cluster architecture. The procedure for choosing gateways is summarized in Table 3.

**Figure 1.**
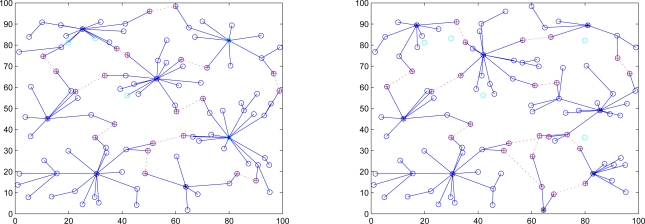
The influence of anti-nodes (cyan) ; the sensor network without secure topology control (left), the sensor network with secure topology control (right).

The goal of Phase I is to guard against anti-nodes hiding in the network and to identify the normal nodes. This is very important because if the anti-nodes participate in the cluster construction process, we can expect that the network operation would be heavily crippled. Figure 1 shows the network topologies of a sensor network with and without secure topology control. With five anti-nodes (cyan) randomly deployed in the field, Figure 1 (left) depicts that anti-nodes play the roles of two clusterheads and three member nodes without secure topology control. On the other hand, with secure topology control, Figure 1 (right) shows that anti-nodes have been intentionally eliminated and then the network members are normal nodes. Hence, the anti-nodes may not affect the network during the cluster forming process.

### 3.3. Phase III: Key Distribution

**Figure 2.**
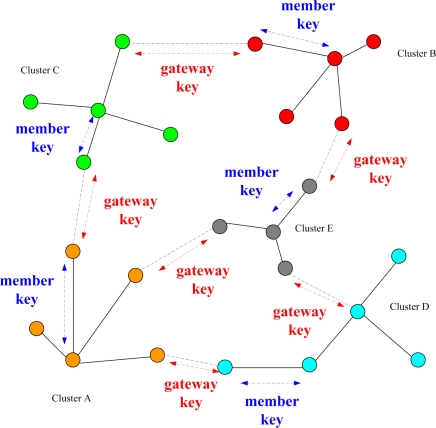
Phase III: Key distribution for WSNs.

According to the cluster construction in Phase II, a simple and efficient key distribution scheme is applied in the network. In this phase, two symmetric shared keys, a cluster key and a gateway key, are encrypted by the pre-distributed key and are distributed locally. A cluster key is a key shared by a clusterhead and all its cluster members, which is mainly used for securing locally broadcast messages, e.g., routing control information, or securing sensor messages. Moreover, in order to form a secure communication channel between the gateways of adjacent clusters, a symmetric shared key may be used to encrypt the sending message. The process of key distribution is shown in Figure 2. In this phase, another challenge may be made to guard against anti-nodes that have not been found out in Phase I. The challenge is that if any sensor cannot decrypt ciphertext encrypted by a cluster key or a gateway key, the node will be removed from the member or neighbor list. Therefore, the security of intra-cluster communication and inter-cluster communication are established upon a cluster key and a shared gateway key, respectively.

### 3.4. Phase IV: Key Renewal

Using the same encryption key for extended periods may incur a cryptanalysis risk. To protect the sensor network and prevent the the adversary from getting the keys, key renewing may be necessary. In the case of the revocation, in order to accomplish the renewal of the keys, the originator node generates a renewal index, and forwards the index to the gateways. The procedures of key renewal are detailed as follows.

**Figure 3.**
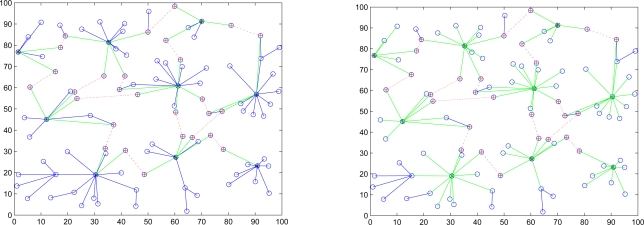
Key renewal process: the originator sends the renewal index to other clusterheads through gateways (left); the clusterheads send the renewal index to their cluster members (right).

**Figure 4.**
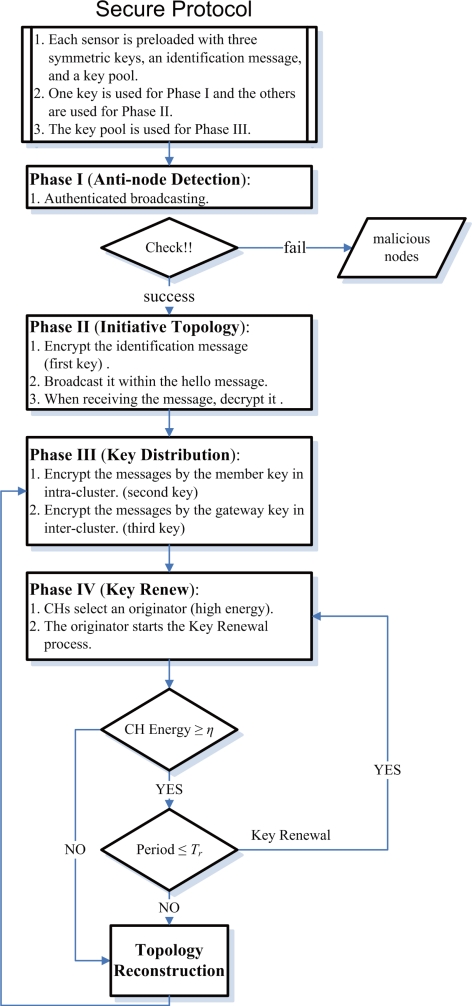
Secure distributed topology control for WSNs.

Initially all clusterheads (CHs) choose an originator to start the “key renewals”, and then it will send the index to all clusterheads in the network. There are many possible approaches for determining the originator. For instance, the clusterhead with the highest energy level or the clusterhead with the lowest cluster ID. After selecting the originator, it initializes the “Key renewal” process and sends the index to its neighboring clusters by gateways. Then the clusterhead refreshes the two keys from the key pool and broadcasts the two new keys to their cluster members locally. The operation repeats the way through to all clusters in the network. The key renewing process is depicted in Figure 3. A period of time (*T_r_*) is set in order to avoid that the originator does not start the “key renewal” process. If the other clusters do not receive the index after *T_r_*, they will choose a new originator from themselves. The method helps to rescue when the previous originator is broken off. The focal procedures of the SADTCA are summarized in Figure 4.

## 4. Determining the Quarantine Region

If the anti-nodes are scattered randomly in the first deployment of a sensor network, the anti-nodes can be detected by authentication in Phase I of the SADTCA. On the other hand, given the cluster-based topology formed in Phase II of the SADTCA, the clusterhead and cluster members may detect external attacks and check the unsolicited messages by observing the abnormal behaviors of the sending nodes. For instance, filtering the content of the incoming messages, detecting the frequency of the faulty messages, or checking the sending frequency of messages. Thus, these scenarios may imply a possible spam attack and then the clusterhead may broadcast a message throughout the whole cluster to announce the existence of anti-nodes. Therefore, in order to defend against spam attacks, three distributed methods, Method 1: quarantine for clusters, Method 2: quarantine for nodes, and Method 3: quarantine for infected areas, are proposed for dynamically determining the quarantine region.

### 4.1. Method 1: Quarantine for Clusters

When the clusterhead finds out the occurrence of a spam attack, it broadcasts a message throughout the whole cluster. In this condition, the set of quarantine nodes is composed of the clusterhead and cluster members. Note that the performance of the SADTCA with Method 1 may be considered as a conservative approach for forming the quarantine region.

### 4.2. Method 2: Quarantine for Nodes

In this scheme, the quarantine region is the region where the transmission of the anti-node can be received. Thus, the transmission range of an anti-node may be denoted as the distance between the anti-node and the borderline of the quarantine region. The concept of this method is the same as the DASA scheme. However, if the quarantined node is a clusterhead, the whole cluster will be quarantined since clusterheads are important nodes for controlling the cluster operation. On the other hand, if the quarantined node is a cluster member, the whole cluster will not be quarantined.

### 4.3. Method 3: The Infected Areas

**Figure 5.**
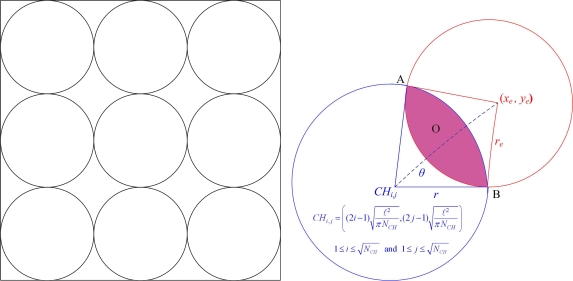
The geometric illustration of cluster distribution; an ideal distribution of clusters (left), the infected region **O** of a cluster (right).

Here we introduce a way to determine the set of quarantine nodes and quarantine region with a threshold of the infected percentage of cluster coverage. Assuming the uniform distribution of the sensor nodes, the clusters may be located one by one from the coordinate of (0,0) in *X-Y* plane as shown in Figure 5 (left). Thus, a decision for quarantine region may be made with proper settings for the normal clusters and anti-nodes.

Since each cluster is responsible for sensing the scope in the network ℓ^2^*/N_CH_*, the possible coverage range of a cluster is

(2) $$r=\sqrt{\frac{\ell^{2}}{\pi N_{\mathrm{CH}}}}$$

where ℓ is the side length of the sensing square and *N_CH_* is the number of clusters. Accordingly, the coordinates of the clusters yields

(3) $$(x_{i},\;y_{j})=((2i-1)r,\;(2j-1)r)=\left((2i-1)\sqrt{\frac{\ell^{2}}{\pi N_{\mathrm{CH}}}}(2j-1)\sqrt{\frac{\ell^{2}}{\pi N_{\mathrm{CH}}}}\right)$$

where $1\leq i\leq \sqrt{N_{\mathrm{CH}}}$ and $1\leq j\leq \sqrt{N_{\mathrm{CH}}}$. Assume the coordinates and the transmission range of an anti-node are (*x_e_*, *y_e_*) and *r_e_*, respectively. As depicted in Figure 5 (right), the infected region O between the coverage of a neighboring clusterhead and an anti-node is given by

(4) $$O=\pi \left(r^{2}\;\left[\frac{{\text{sin}}^{-1}(\frac{\bar{\mathrm{AB}}}{r})}{360}\right]+r_{e}^{2}\;\left[\frac{{\text{sin}}^{-1}(\frac{\bar{\mathrm{AB}}}{r_{e}})}{360}\right]\right)-\frac{\bar{\mathrm{AB}}}{2}\left(r\;\text{cos}\;\left[{\text{sin}}^{-1}(\frac{\bar{\mathrm{AB}}}{2r})\right]+r_{e}\;\text{cos}\;\left[{\text{sin}}^{-1}(\frac{\bar{\mathrm{AB}}}{2r_{e}})\right]\right)$$

where $\bar{\mathrm{AB}}$ is the length between two intersection points ***A*** and ***B***. Therefore, given the infected region **O** and a threshold of infected percentage of the cluster coverage *η*, the decision of quarantine region may be determined. For instance, when

(5) $$O\geq \eta \cdot (\pi r^{2})$$

the infected cluster is quarantined.

Accordingly, when **O**/π*r*^2^ ≥ η, Method 1 may be applied; otherwise, Method 2 may be chosen to quarantine the whole cluster. Therefore, Method 3 achieves the operation balance of Methods 1 and 2 for establishing local quarantine regions.

## 5. Performance Analysis

This section analyzes the performance of the proposed SADTCA scheme. We focus on the increase of communication overheads for data dissemination when facing the spam attack. In order to simplify the analysis, assume that the sensors are uniformly distributed. Established upon the dimension of the infected area, the quarantine region may be determined for the infected cluster as detailed in Section 4.. Furthermore, the average hop difference between the information routing by the shortest path without considering the quarantine region and the routing by the shortest path avoiding the quarantine region is derived in Section 5.1.

### 5.1. The Routing Variation

**Figure 6.**
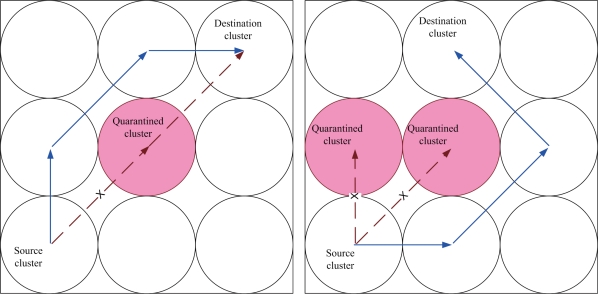
The path interference of the quarantined cluster; one quarantined cluster (left), two quarantined clusters (right).

**Figure 7.**
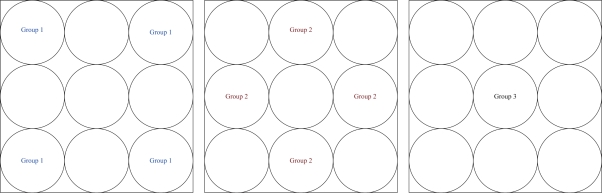
Three possible locations of the source; group 1 (left), group 2 (middle), group 3 (right).

Given one anti-node in the network, the shortest routing path may be interfered by the quarantined clusters. Such interference leads to the extra routing hops, which is demonstrated in Figure 6. The impact of the quarantine region on routing performance is investigated by calculating the routing variation for sending one message from one cluster to another cluster (normalized). Let the routing variation $E[N_{\text{hop}}^{\left(k\right)}]$ be the hop difference between the shortest path for routing with quarantine region and without quarantine region of source cluster *k*. Suppose that anti-nodes are mutually independent and the transmission range of anti-nodes is the same as normal nodes. Since $E[N_{\text{hop}}^{\left(k\right)}]$ is related to the source, the number and location of quarantine cluster, and the destination, referring to Figure 7, the locations of source clusters are classified into three possible groups. Hence, the normalized routing variation $E[N_{\text{hop}}^{\left(k\right)}]$ may yield

(6) $$E[N_{\text{hop}}^{\left(k\right)}]=\sum_{i=1}^{3}\sum_{j=0}^{m}P_{j}^{\left(k,i\right)}\cdot E[N_{j}^{\left(k,i\right)}]$$

where $P_{j}^{\left(k,i\right)}$ is the probability of *j* quarantine clusters with source cluster *k* belonging to group *i*, $E[N_{j}^{\left(k,i\right)}]$ is the routing variation with *j* quarantined clusters with source cluster *k* belonging to group *i*, and *m* is the maximal value of the quarantined clusters. Thus, the normalized routing variation $E[N_{\text{hop}}^{1}]$ with one anti-node is given by

(7) $$E[N_{\text{hop}}^{1}]=\frac{1}{N_{\mathrm{CH}}}\cdot \sum_{k\in I}\sum_{i=1}^{3}\sum_{j=0}^{m}P_{j}^{\left(k,i\right)}\cdot E[N_{j}^{\left(k,i\right)}]$$

where *I* is the index set of clusterheads.

**Figure 8.**
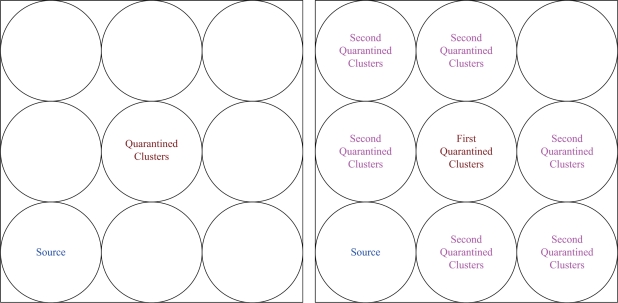
The locations of quarantined clusters given the location of source group 1; one cluster quarantined (left), two clusters quarantined with possible locations of the second quarantined cluster (right).

**Figure 9.**
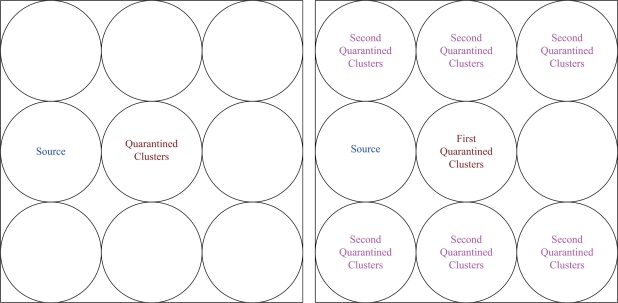
The locations of quarantined clusters given the location of source group 2; one cluster quarantined (left), two clusters quarantined with possible locations of the second quarantined cluster (right).

**Figure 10.**
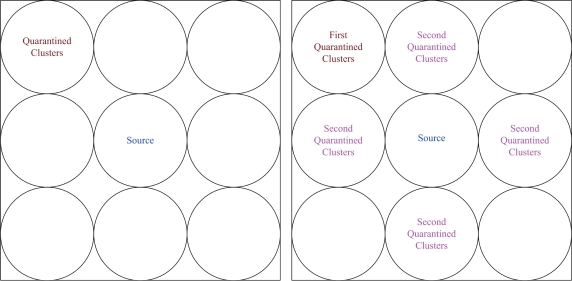
The locations of quarantined clusters given the location of source group 3; one cluster quarantined (left), two clusters quarantined with possible locations of the second quarantined cluster (right).

Referring to Figure 8 for group 1, Figure 9 for group 2, Figure 10 for group 3, and with the infected percentage of the cluster coverage *η* = 1/3, the normalized routing variation $E[N_{\text{hop}}^{1}]$ is

(8) $$E\left[N_{\text{hop}}^{1}\right]≃0.0965$$

from experimental analysis. Similarly, assuming *η* is 1/5, we obtain

(9) $$E[N_{\text{hop}}^{1}]≃0.1815$$

Given that the sensors are uniformly distributed with high network density, the anti-nodes may be considered as mutually independent. Thus, the normalized routing variation *E*[*N*_hop_] with *Q* anti-nodes yields

(10) $$E[N_{\text{hop}}^{Q}]=Q\cdot E[N_{\text{hop}}^{1}]$$

which depicts the difference between the number of hops of the shortest path for routing with quarantine region and that for routing without quarantine region.

### 5.2. Analysis of Energy Consumption

This subsection analyzes the energy consumption of the SADTCA when executing the following three phases: cluster formation (Phase II), key distribution (Phase III), and key renewal (Phase IV). The total power requirements include both the power required to transmit and to receive (or process) messages.

#### Phase II: Clusterhead Selection

The energy consumption of clusterhead selection assuming homogenous sensors is examined. In the initialization phase, each sensor broadcasts a *Hello* message to its neighboring sensors. Therefore, the number of transmissions *N_T_x__* is equal to the number of sensors in the network, *n*, and the number of receptions *N_R_x__* is the sum of the neighboring sensors of each sensor. That is,

(11) $$N_{T_{x}}=n,\;\;\text{and }\;N_{R_{x}}=\sum_{j=1}^{n}N_{j}$$

where *N_j_* is the number of neighboring sensors of sensor *j*.

As a sensor, say sensor *i*, meets the conditions of being a clusterhead, it broadcasts this and assigns cluster ID *i* to its neighboring sensors. Its neighboring sensors then transmit a signal to their neighbors to update cluster ID information. During this clustering phase, (1+*N_i_*) transmissions and (*N_i_* + ∑ _*j*∈*C*_*i*__ *N_j_*) receptions are executed, where *C_i_* is the index set of neighboring sensors of sensor *i*. This procedure is applied to all clusterheads and their cluster members. Now let $N_{T_{x}}^{c}$ and $N_{R_{x}}^{c}$ denote the number of transmissions and receptions for all clusters, respectively. Hence,

(12) $$N_{T_{x}}^{c}=\sum_{i\in I}\left(1+N_{i}\right)$$

(13) $$N_{R_{x}}^{c}=\sum_{i\in I}(\sum_{j\in C_{i}}N_{j}+N_{i})$$

where *I* is a index set of clusterheads. Therefore, the total number of transmissions *N_T_* and the number of receptions *N_R_* are

(14) $$N_{T}=N_{T_{x}}+N_{T_{x}}^{c}=n+\sum_{i\in I}\left(1+N_{i}\right)$$

(15) $$N_{R}=N_{R_{x}}+N_{R_{x}}^{c}=\sum_{j=1}^{n}N_{j}+\sum_{i\in I}(\sum_{j\in C_{i}}N_{j}+N_{i})$$

Suppose that the energy needed for the transmission is *E_T_*, which depends on the transmitting range *R*, the energy needed for the reception is *E_R_*, the energy needed for the encryption is *E_enp_*, and the energy needed for decryption is *E_dep_*. From (24) and (25), the total energy consumption, *E_total_*, for cluster formation in the wireless sensor network is

(16) $$E_{\mathrm{total}}=N_{T}\cdot (E_{T}+E_{\mathrm{enp}})+N_{R}\cdot (E_{R}+E_{\mathrm{dep}})$$

Observe that the above analysis is suitable for any transmitting range. However, overly small transmission ranges may result in isolated clusters whereas overly large transmission ranges may result in a single cluster. Therefore, in order to optimize energy consumption and encourage linking between clusters, it is more reasonable to consider the minimum transmission power (or range *R*) which will result in a fully connected network.

#### Phase III: Key Distribution

In order to simplify the presentation, the main notations are introduced as follows: let *I* denote the index set of clusterheads; let *H* denote the index set of 1-hop cluster members in the network; let *H_i_* denote the index set of 1-hop cluster members of cluster *i* (a subset of *H*); let *M* denote the index set of 2-hop cluster members in the network; let *M_i_* denote the index set of 2-hop cluster members of cluster *i* (a subset of *M*); similarly, let *S* be the index set of sensors neighboring with 2-hop cluster members; let *S_i_* be the index set of sensors neighboring with 2-hop cluster members of cluster *i* (a subset of *S*); let *G* be the index set of gateway nodes.

In this phase, Diffie-Hellman key exchange is used when setting the gateway key and *E_pro_* is the consumed energy of Diffie-Hellman key exchange. When clusterheads broadcast messages to trigger the key distribution procedure, the number of transmission *D_T_* and reception *D_R_* can be expressed by

(17) $$D_{T}=\sum_{i\in I}\sum_{j\in S_{i}}N_{j}+\left|I\right|+\left|G\right|$$

(18) $$D_{R}=\sum_{i\in I}\sum_{j\in H_{i}}N_{j}+\sum_{i\in I}\sum_{j\in M_{i}}N_{j}+\left|G\right|$$

Thus, based on the energy needed to transmit and receive, the total energy consumption for key distribution yields:

(19) $$E_{\mathrm{key}}=D_{T}E_{T}+D_{R}E_{R}+\left|G\right|E_{\mathrm{pro}}$$

#### Phase IV: Key Renewal

In a unicast-based group rekeying, the communication complexity is *O*(*N*), where *N* is the group size. Logical key trees can be used to reduce the complexity of group rekeying schemes (from *O*(*N*) to *O*(*logN*) [6]) and to enhance the scalability of group rekeying operation. Hence, based on a logical key tree, the communication cost of a group rekeying in the SADTCA is *O*(*logN*).

### 5.3. Comparison of the SADTCA and the DADS

This subsection considers the energy consumption for forming a quarantine region with the proposed SADTCA scheme and the DADS [17] when facing the spam attack.

#### The DADS

For the DADS scheme, denote *d_q_* as the distance between the anti-node and the borderline of the quarantine region. Here we consider two scenarios, *d_q_* = *R* and *d_q_* = 2*R*, where *R* is the transmission range of a sensor node. The first scenario considers that an anti-node threatens the neighboring sensor nodes that are of *d_q_* = *R*. The second scenario considers that an anti-node threatens the whole cluster. Since the network infrastructure of the SADTCA is based on 2-hop cluster topology, the DASA scheme with *d_q_* = 2*R* may be used to benchmark the performance of the proposed SADTCA with Method 1 (quarantine for clusters).

For the DADS scheme with *d_q_* = *R*, the total energy consumption for determining the set of quarantine nodes is

(20) $$E_{\mathrm{total}}^{\left(R\right)}=N_{T}^{\left(R\right)}\cdot (E_{T}+E_{\mathrm{enp}})+N_{R}^{\left(R\right)}\cdot (E_{R}+E_{\mathrm{dep}})$$

where the total number of transmissions $N_{T}^{\left(R\right)}$ and the number of receptions $N_{R}^{\left(R\right)}$ are

(21) $$N_{T}^{\left(R\right)}=N_{a}\;\;\text{and }\;\;N_{R}^{\left(R\right)}=N_{a}+\sum_{i\in B_{a}^{\left(R\right)}}N_{i}$$

Note that *N_a_* is the number of neighboring sensors of the anti-node and $B_{a}^{\left(R\right)}$ is the index set of sensors neighboring with the anti-node.

Similarly, for the DADS scheme with *d_q_* = 2*R*, considering the authentication phase and the quarantine region, the total number of transmissions $N_{T}^{\left(2R\right)}$ and the number of receptions $N_{R}^{\left(2R\right)}$ may be approximated by

(22) $$N_{T}^{\left(2R\right)}=2N_{a}$$

(23) $$N_{R}^{\left(2R\right)}=N_{a}+2\sum_{i\in B_{a}^{\left(2R\right)}}N_{i}$$

where $B_{a}^{\left(2R\right)}$ is the index set of sensors neighboring with the anti-node. Thus, the total energy consumption yields $E_{\mathrm{total}}^{\left(2R\right)}=N_{T}^{\left(2R\right)}\cdot (E_{T}+E_{\mathrm{enp}})+N_{R}^{\left(2R\right)}\cdot (E_{R}+E_{\mathrm{dep}})$

#### The SADTCA

For the SADTCA scheme, three distributed methods, Method 1: quarantine for clusters, Method 2: quarantine for nodes, and Method 3: quarantine for infected areas, are examined for dynamically determining the quarantine region.

In Method 1, the set of quarantine nodes is composed of the clusterhead and cluster members. Assume cluster *k* is attacked by an anti-node. Thus, the total number of transmissions *N_T_* and the number of receptions *N_R_* are

(24) $$N_{T}=1+N_{a}+\left|M_{k}\right|$$

(25) $$N_{R}=N_{\mathrm{ch}}+2\sum_{i\in M_{k}}N_{i}+\sum_{i\in B_{a}}N_{i}$$

where *M_k_* is the index set of 2-hop cluster members of cluster *k*, *B_a_* is the index set of sensors neighboring with the anti-node, *N_a_* is the number of neighboring sensors of the anti-node, and *N_ch_* is the number of neighboring sensors of the clusterhead. The energy consumption in a cluster is $E_{\mathrm{total}}^{\left(\mathrm{ch}\right)}=N_{T}\cdot (E_{T}+E_{\mathrm{enp}})+N_{R}\cdot (E_{R}+E_{\mathrm{dep}})$. Since the quarantine nodes may belong to different clusters, the total energy consumption yields

(26) $$E_{\mathrm{total}}=N_{c}\cdot E_{\mathrm{total}}^{\left(\mathrm{ch}\right)}$$

where *N_c_* is the number of neighboring clusters of the anti-node.

In Method 2, assuming that a clusterhead is not quarantined, the SADTCA scheme consumes the same energy as the DADS scheme with *d_q_* = *R* without using the information of cluster topology (as described in (20)).

In Method 3, the proposed SADTCA introduces a way to determine the set of quarantine nodes and quarantine region with a threshold of the infected percentage of cluster coverage *η* (as detailed in Section 4.3.). Thus, when **O**/π*r*^2^ *< η*, the energy consumption can be described by (20); otherwise, we may use (26) to represent the energy consumption for quarantining the whole cluster.

Based upon the above analysis, the energy consumptions of the proposed quarantine strategies are comparable to that of the DADS scheme. The comparison of the percentage of quarantine region in the sensing field with the proposed quarantine methods and the DADS scheme is described in Section 6.4..

## 6. Simulation

**Figure 11.**
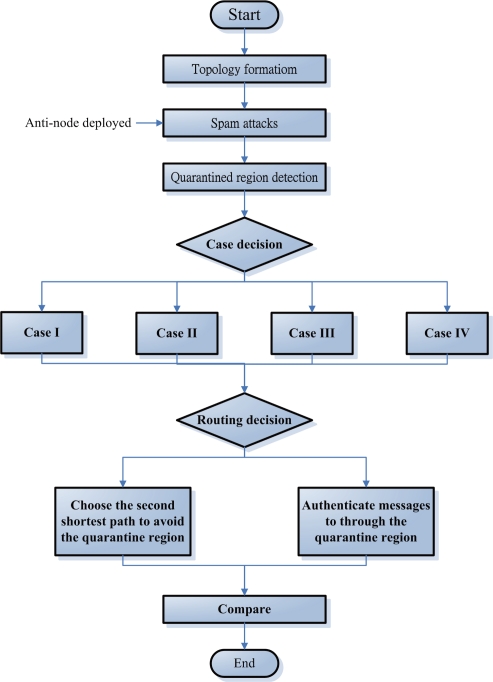
The simulation flowchart of the SADTCA scheme.

In this section, we study the performance of the proposed SADTCA scheme via simulation when executing the four cases: quarantine for clusters, quarantine for nodes, quarantine with *η* ≥ 1/3, and quarantine with *η* ≥ 1/5 (as detailed in Section 4.). Referring to [18], the simulation flow chart is shown in Figure 11.

**Figure 12.**
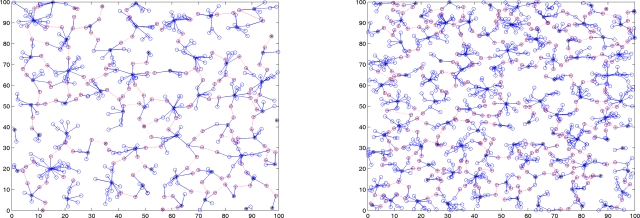
A random network with 500 sensors, *R* = 6.33, and ℓ = 100 (left); a random network with 1000 sensors, *R* = 4.48, and ℓ = 100 (right).

For the experiments, 100, 500, 1000 (Figures 12) sensor nodes are randomly deployed in the sensor field 100×100 units in size. In order to maintain the network connectivity with high probability, the transmitting range *R* of sensors may be given by [21]:

(27) $$R\approx \ell \sqrt[{d}]{\frac{\text{log}\ell }{n}}$$

where *n* is the number of sensors and ℓ is the length of sides of a *d*-dimensional cube.

Here we focus on two kinds of variation. One is the hop difference between the shortest path through the quarantine region and the shortest path avoiding the quarantine region. The other is the number of hops with authenticated messages through the quarantine region. We assume that each cluster sends one message to other clusters, except the quarantined clusters, and then calculate the total number of hops.

### 6.1. Case I: Quarantine for Clusters

**Figure 13.**
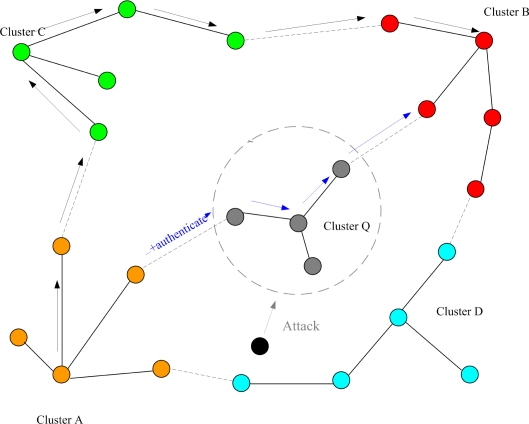
Authentication of quarantine process for clusters (Case I).

**Figure 14.**
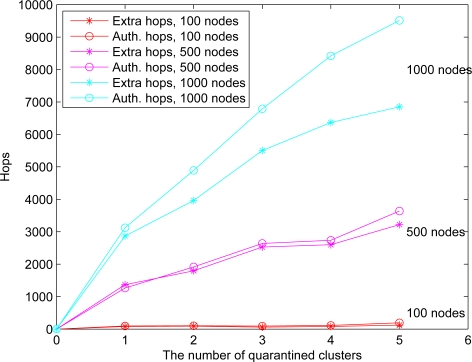
Case I: the number of extra hops for bypassing routing path and the number of authenticated hops for going through the quarantined clusters.

This set of experiments investigates the performance of the quarantine strategy for clusters with varying the number of anti-nodes ranging from 1 to 5. We assume that the attacker threatens different clusters. If the cluster is attacked, it will be quarantined. A description of quarantine process for clusters is illustrated in Figure 13. As depicted in Figure 14, the number of extra hops for bypassing routing path is less than the number of authenticated hops for going through the quarantined clusters. Thus, a bypassing routing may be efficient in this case.

### 6.2. Case II: Quarantine for Nodes

**Figure 15.**
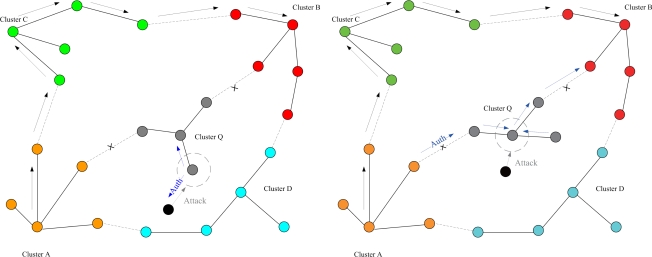
Authentication of quarantined nodes (Case II): a member node (left) and a clusterhead (right).

**Figure 16.**
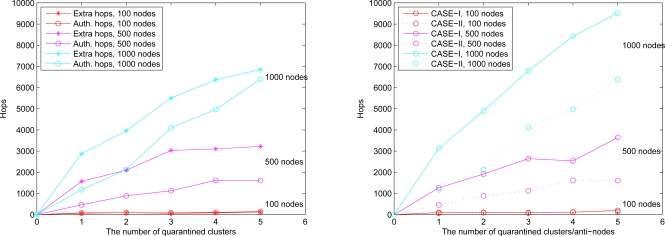
Case II: the number of extra hops for bypassing routing path and the number of authenticated hops for going through the quarantined clusters (left); the comparison of authenticated hops of Case I and Case II (right).

In Case II, we assume that the attacker threatens the nodes. If the node is attacked, it will be quarantined. When the quarantined node is a member, the authentications are executed between the quarantined node and the clusterhead, and between the quarantined node and the attacker since it may prevent the threat from extending to the whole cluster. In this scenario, a message is allowed to pass through the cluster. A description of quarantine for nodes is shown in Figure 15 and the result is depicted in Figure 16, which compares the total number of hops with authenticated messages through the quarantine region for Case I and Case II, respectively. Figure 16 shows that the number of authenticated hops for going through the infected clusters is less than the number of extra hops for bypassing the routing path. Hence, a bypass routing may not be efficient in this case. Observe that the number of hops in Case II is much less than that in Case I. Therefore, Case II may have a better energy control than Case I.

### 6.3. Quarantine for Infected Areas (Cases III and IV)

When anti-nodes start spam attacks, the quarantine region of the infected cluster can be determined based on the dimension of infected area (**O**) as detailed in Section 4. In Case III, if the dimension ratio of infected area (**O**) to the cluster coverage is over 1/3, the cluster will be quarantined. Similarly, in Case IV, if the dimension ratio of infected area (**O**) to the cluster coverage is over 1/5, the cluster will be quarantined. Assuming the sensors are uniformly distributed, instead of using the criterion in (5), the ratio of the number of nodes within the transmission range of an anti-node to the number of nodes within a cluster sensing scope (*i.e.*, the number of infected cluster members) may be applied to determine the quarantine region. Experimental results show that this ratio can well represent the cover ratio in a random network with high network density.

**Figure 17.**
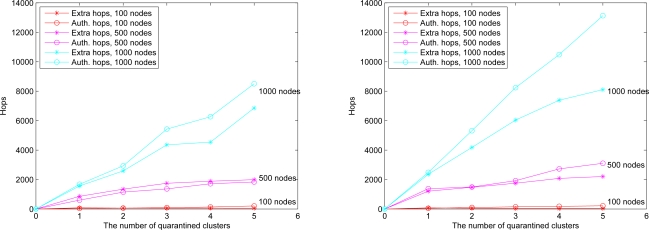
The number of extra hops for bypassing routing path and the number of authenticated hops for going through the quarantined clusters: the result of **O** ≥ 1/3 (Case III) (left), the result of **O** ≥ 1/5 (Case IV) (right).

**Figure 18.**
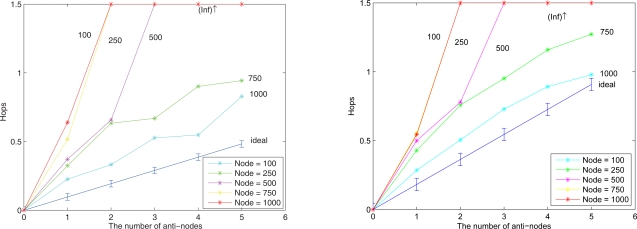
Average extra hops for bypassing routing path and the ideal line of **O** ≥ 1/3 (Case III) (left), the result and the ideal line of **O** ≥ 1/5 (Case IV) (right).

We assume an anti-node’s transmission range is the same as normal nodes. The results of Case III and Case IV are depicted in Figure 17 (left) and Figure 17 (right), respectively. Figure 17 shows that compared with the scheme in Case III, the mechanism in Case IV is more secure to defend against spam attacks, but with higher energy consumption. Figure 18 depicts that with a larger network scale, the performance of the proposed approach matches well with the derived ideal results. Note that the (Inf) in this figure indicates that a bypassing routing path is not reachable due to the low density of nodes.

### 6.4. Proportion of the Quarantine Region

The percentage of the quarantine region in the sensing field impacts two performance metrics of sensor networks: network lifetime and network connectivity. This is because the message authentication in quarantine region represents extra energy consumption of sensors, which may shorten the lifetime of sensor networks. Moreover, with a high percentage of quarantine region in the network, a sensing field might be partitioned due to the energy depletion of infected sensor nodes. Notice that the quarantine region proportion reflects the control of infected nodes within the neighborhood of an attack.

**Figure 19.**
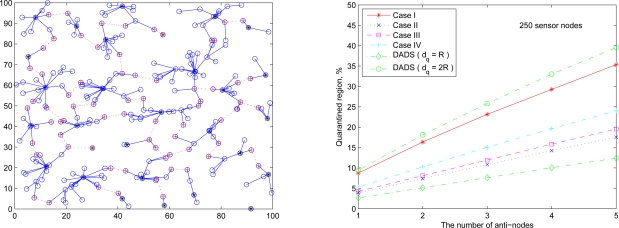
A random network of 250 sensors deployed based on uniform distribution (left); the average percentage of quarantine region with R = 9.4 (right).

Referring to the transmission range of sensors given by (27), Figure 19 (left) and Figure 19 (right) show the cluster topology and the average percentage of the quarantine region with 250 sensor nodes using different quarantine methods, respectively. The quarantine strategy for clusters (Case I) stands for the most strict quarantine condition and the quarantine strategy for nodes (Case II) stands for the loosest one. In the worst case (Case I), 5 anti-nodes cause about 35% of the entire network to be marked as the quarantine region. Thus, only about 35% of the network nodes need to authenticate the messages, which means the 65% of network nodes do not pay the cost for authentication. The quarantine region proportion of Case II is about half of the proportion of quarantine region in Case I (about 17%). The quarantine strategies for infected areas (Cases III and IV) can reduce the average percentage to 24% (Case IV) and even 19% (Case III).

Observe that, as shown in Figure 19 (right), the performance of the DADS with *d_q_* = *R* may represent a lower bound for the performance of the SADTCA with the quarantine strategies. Due to the 2-hop cluster topology, the quarantine strategy for clusters (Case I) expands the quarantine region, which makes the average percentage of Case I close to that of the DADS with *d_q_* = 2*R*. Thus, the DASA scheme with *d_q_* = 2*R* may be used to benchmark the performance of the proposed SADTCA with Method 1 (*i.e.*, Case I: quarantine for clusters).

**Figure 20.**
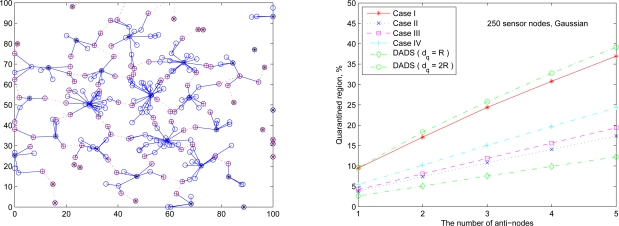
A random network of 250 sensors deployed based on Gaussian distribution with sensor deployment strategy I (left); the average percentage of the quarantine region (right).

**Figure 21.**
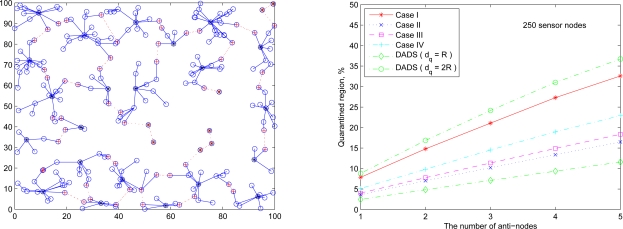
A random network of 250 sensors deployed based on sensor deployment strategy II (left); the average percentage of the quarantine region (right).

The following set of experiments investigates the influence of the distribution of sensor nodes on the proportion of quarantine region. Here two sensor deployment strategies are considered: (I) Making sensor density high at the center of the terrain, and (II) Making sensor density high at the border of the terrain. For deployment strategy I, Figure 20 (right) shows the average percentage of the quarantine region assuming that the 250 sensors are deployed based on Gaussian distribution with the center (*x*_0_, *y*_0_) = (50, 50) and the spreads of the blob σ*_x_* = σ*_y_* = 0.25ℓ (Figure 20 (left)). For deployment strategy II, assuming that 50 sensors are deployed within the center sensing field 80 × 80 units in size and the other 200 nodes are deployed outside the center square (Figure 21 (left)), Figure 21 (right) shows the proportion of the quarantine region in the sensing field. Observe that these performances are similar to the one with uniform distribution as shown in Figure 19 (right). Thus, except under extreme conditions for specific topologies, the distribution of the sensor nodes may not have significant impact on the performance of the proposed quarantine strategies.

In a flat network topology, the DADS scheme considers the neighborhood control of an attack and provides a heuristic way to determine the quarantine region. On the other hand, in a hierarchical network topology, the SADTCA explores the cluster structure and applies distributed quarantine strategies to determine the set of infected nodes. Although the DADS has a less proportion of the quarantine region, by adjusting the threshold of infected percentage of the cluster coverage η, our schemes can dynamically coordinate the proportion of the quarantine region and adaptively achieve the cluster control and the neighborhood control of attacks.

### 6.5. Energy Consumption

**Figure 22.**
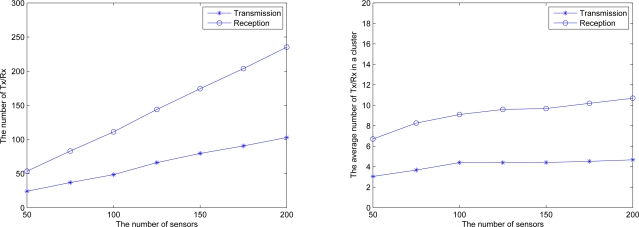
Communications of key distribution process; total number of transmission and reception in the network (left), the average number of transmission and reception in a cluster (right).

Figure 22 (left) illustrates the total number of transmission and reception in the network for executing key distribution, which increases with the increasing number of sensors. Figure 22 (right) illustrates the average number of transmission and reception in a cluster for executing key distribution. Observe that with a sensible topology control in Phase II, the average number of transmission and reception in a cluster increases slightly when the number of sensors increases.

## 7. Conclusions

We describe a secure protocol for topology management in wireless sensor networks. By adaptively forming quarantine regions, the proposed secure protocol is demonstrated to reach a network security agreement and can effectively protect the network from energy-exhaustion attacks. Therefore, in a hierarchical network topology, the SADTCA scheme can adapt cluster control and neighborhood control in order to achieve dynamic topology management of the spam attacks. Compared with the DADS scheme, our protocol can be used to enhance the control of quarantine region, as well as to restrain the number of packet transmissions caused by anti-nodes.

Although the initial secure goals of the research have been achieved in this paper, further experimental and theoretical extensions are possible. In our future work, we plan to involve more mechanisms to make the protocol faultless and practical, such as developing a new algorithm to identity anti-network sensors and proposing efficient security mechanisms to make protocol suitable for adaptive topology management.

## Figures and Tables

**Table 1.** Secure Cluster Formation.

1. Each sensor initializes a random waiting timer with a value $WT_{i}^{\left(0\right)}$.
2. Each sensor encrypts the *Plaintext* with the *Hello* message.
3. Each sensor transmits the *Hello* message at random times: Draw a sample *r* from the distribution $\lambda \cdot WT_{i}^{\left(0\right)}\cdot U(0,1)$, where 0 < λ < 0.5 wait *r* time units and then transmit the *Hello*.
4. Each sensor receives the *Hello* message and decrypts it.
**if** the decrypted *Ciphertext* is the same as the preload message
the sensor is a normal node.
**else**
(a) the sensor is an anti-node.
(b) it should be removed from the neighbor list.
**end**
5. Establish and update the neighbor identification:
**if** a sensor receives a message of assigning a cluster ID at time step *k*
(a) join the corresponding cluster.
(b) draw a sample *r*′ from the distribution $WT_{i}^{\left(k\right)}\cdot U(0,1)$.
(c) wait *r*′ time units and then send an updated *Hello* message with the new cluster ID.
(d) stop the waiting timer. (Stop!)
**else**
collect neighboring information.
**end**
6. Decrease the random waiting time according to equation (1).
7. Clusterhead check:
**if***WT_i_* = 0 and the neighboring sensors are not in another cluster
(a) broadcast itself to be a clusterhead.
(b) assign the neighboring sensors to cluster ID *i*. (Stop!)
**elseif***WT_i_* = 0 and some of the neighboring sensors are in other clusters
stand by. (Stop!)
**else**
go to Step 3.
**end**

**Table 2.** Description of the Counter Initialization Process.

**while** (sensor *n_i_* is a neighboring sensor of *m_j_*)
**if***n_i_* is a clusterhead
$C_{ij}^{\left(n_{i}\right)}=C_{ij}^{\left(n_{i}\right)}+10\beta$
**else**
$C_{ij}^{\left(n_{i}\right)}=C_{ij}^{\left(n_{i}\right)}+\beta$
**end**
**end**
where $C_{ij}^{\left(n_{i}\right)}$ is the counter of sensor *n_i_* for cluster *j*, $\beta =\alpha (1-\frac{d_{n_{i}m_{j}}}{R})$ with a positive integer *α*, *d_n_i_m_j__* is the distance between sensors *n_i_* and *m_j_*, and *R* is the transmission range.

**Table 3.** Description of Gateway Selection.

a) Based on the cluster formation in Phase I, clusterheads broadcast messages to trigger the gateway selection process.
b) Initialize a vector of random waiting times ${WT}_{ij}^{\left(n_{i},k\right)}$, where ${WT}_{ij}^{\left(n_{i},k\right)}$ is the waiting time of sensor *n_i_* for cluster *j* at time step *k*.
c) Initialize a counter of sensor *n_i_*, $C_{ij}^{\left(n_{i}\right)}$, for gateway selection in cluster *i* to cluster *j*.
d) Decrease the waiting time
${WT}_{ij}^{\left(n_{i},k+1\right)}={WT}_{ij}^{\left(n_{i},k\right)}-C_{ij}^{\left(n_{i}\right)}$.
e) Gateway check:
**if**$WT_{ij}^{\left(n_{i},k\right)}=0$
(1) assign *G_ij_* = *n_i_*, and then
*G_ij_* broadcasts the gateway information to its neighbors.
(2) set $C_{ij}^{\left(x_{i}\right)}=0$ and stop the waiting timer for all neighboring sensors *x_i_* in cluster *i*.
**else**
go to step d).
**end**
